# Molecular Determination of Vascular Endothelial Growth Factor, miRNA-423 Gene Abnormalities by Utilizing ARMS-PCR and Their Association with Fetal Hemoglobin Expression in the Patients with Sickle Cell Disease

**DOI:** 10.3390/cimb44060175

**Published:** 2022-06-01

**Authors:** Abdullah Hamadi, Rashid Mir, Ali Mahzari, Abdulrahim Hakami, Reema Almotairi, Gasim Dobie, Fawaz Hamdi, Mohammed Hassan Nahari, Razan Alhefzi, Mohammed Alasseiri, Nora Y. Hakami, Hadeel Al Sadoun, Osama M. Al-Amer, Jameel Barnawi, Hassan A. Madkhali

**Affiliations:** 1 Department of Medical Laboratory Technology, Faculty of Applied Medical Sciences, University of Tabuk, Tabuk 71491, Saudi Arabia; ralmotairi@ut.edu.sa (R.A.); malasseiri@ut.edu.sa (M.A.); oalamer@ut.edu.sa (O.M.A.-A.); jbarnawi@ut.edu.sa (J.B.); 2 Department of Laboratory Medicine, Faculty of Applied Medical Sciences, Albaha University, Albaha 65431, Saudi Arabia; amoosa@bu.edu.sa; 3 Department of Clinical Laboratory Sciences, Faculty of Applied Medical Sciences, King Khalid University, Abha 61421, Saudi Arabia; ahakami@kku.edu.sa (A.H.); ralhefdy@kku.edu.sa (R.A.); 4 Department of Medical Laboratory Technology, Faculty of Applied Medical Sciences, Jazan University, Jazan 45142, Saudi Arabia; gdobie@jazanu.edu.sa; 5 Department of Clinical Laboratory, Samtah General Hospital, Ministry of Health, Jazan 45142, Saudi Arabia; fhamdi@moh.gov.sa; 6 Department of Laboratory Science, Faculty of Applied Medical Sciences, Najran University, Najran 55461, Saudi Arabia; mhnahari@nu.edu.sa; 7 Department of Medical Laboratory Technology, Faculty of Applied Medical Sciences, King Abdulaziz University, Jeddah 22254, Saudi Arabia; oahakami3@kau.edu.sa (N.Y.H.); hsadounkau.ed.sa@kau.edu.sa (H.A.S.); 8 Department of Pharmacology and Toxicology, College of Pharmacy, Prince Sattam Bin Abdulaziz University, Al-Kharj 16278, Saudi Arabia; h.madkhali@psau.edu.sa

**Keywords:** sickle cell disease-SCD, microRNA-423, SNP-single-nucleotide polymorphism, ARMS-amplification refractory mutation system, OR-odds ratio, CI-confidence interval, fetal hemoglobin HbF, sickle cell disease severity

## Abstract

Recent studies have indicated that microRNA and VEGF are considered to be genetic modifiers and are associated with elevated levels of fetal haemoglobin HbF, and thus they reduce the clinical impact of sickle haemoglobin (HbS) patients. This cross-sectional study was performed on clinical confirmed subjects of SCD cases. miR-423-rs6505162 C>T and VEGF-2578 C>A genotyping was conducted by ARMS-PCR in SCD and healthy controls. A strong clinical significance was reported while comparing the association of miR-423 C>T genotypes between SCD patients and controls (*p* = 0.031). The microRNA-423 AA genotype was associated with an increased severity of SCD in codominant model with odd ratio (OR = 2.36, 95% CI, (1.15–4.84), *p* = 0.018) and similarly a significant association was observed in recessive inheritance model for microRNA-423 AA vs (CC+CA) genotypes (OR = 2.19, 95% CI, (1.32–3.62), *p* < 0.002). The A allele was associated with SCD severity (OR = 1.57, 95% CI, (1.13–2.19), *p* < 0.007). The distribution of VEGF-2578 C>A genotypes between SCD patients and healthy controls was significant (*p* < 0.013). Our results indicated that in the codominant model, the VEGF-2578-CA genotype was strongly associated with increased SCD severity with OR = 2.56, 95% CI, (1.36–4.82), *p* < 0.003. The higher expression of HbA1 (65.9%), HbA2 (4.40%), was reported in SCD patients carrying miR-423-AA genotype than miR-423 CA genotype in *SCD* patients carrying miR-423 CA genotype HbA1 (59.98%), HbA2 (3.74%) whereas *SCD* patients carrying miR-423 CA genotype has higher expression of HbF (0.98%) and HbS (38.1%) than in the patients carrying AA genotype HbF (0.60%), HbS (36.1%). ARMS-PCR has been proven to be rapid, inexpensive and is highly applicable to gene mutation screening in laboratories and clinical practices. This research highlights the significance of elucidating genetic determinants that play roles in the amelioration of the HbF levels that is used as an indicator of severity of clinical complications of the monogenic disease. Further well-designed studies with larger sample sizes are necessary to confirm our findings.

## 1. Introduction

Sickle cell anemia (SCA) is a monogenetic hematological disorder caused by homozygosity mutation in the beta-globin gene on chromosome 11 [1]. SCA was the “first molecular disease”, where technological innovations and chemical differences used to evaluate the abnormality of genes [2]. According to the World Health Organization (WHO), 5.2% of the world’s population is affected by SCD and this percentage varies in Saudi Arabia’s population according to provinces and areas [3]. The Saudi population is considered at high risk of suffering from SCA due to traditional, cultural, and social factors [2,4]. Importantly, there are several causes for clinical and hematologic variability in SCA patients including the single mutation in the beta-globin gene, environmental effects and other genetic modifiers [5,6]. With modern molecular techniques and advanced genotype testing, there is insufficient information about nucleotide polymorphisms analysis and frequency for SCA patients in Saudi Arabia [7].

An earlier study investigated the relationship between genetic polymorphism and sickle cell mutation [8]. The miR-423 exists in 2 mature forms called miR-423-3p and miR-423-5p and their modified expression has been documented in several hematological disorders and cancers [9,10,11,12]. Current studies have indicated that microRNAs play crucial role in the differentiation and maturing of RBC, expressing hematopoietic factors and thereby regulating the expression of globin genes via post-transcriptional gene silencing and there significant change in the microRNAs (miR-320, miR-144, miR-451, miR-503) expression in/sickle and thalassemic cells compared with normal RBCS and leads to clinical severity [13]. Research scientists are trying to develop some new strategies for increasing HbF induction such as identifying new molecular targets that regulate γ-globin gene transcription and translation [14].

Primary erythroid progenitor data and genome wide miRNA microarray support the fact that indicates a γ-globin gene regulation in sickle cell disease is regulated by miR-144/NRF2-mediated mechanism [15]. Taken together, it has been concluded that microRNAs in erythrocytes may act as a genetic modifier of HBS in Sickle cell anemia and may exhibit innovative insights into the clinical pathobiology heterogeneity of sickle cell disease [16]. MicroRNAs is reported to control the translation of many genes involved in erythropoiesis. However, limited or no research studies have documented the link between miRNA-423C>A gene polymorphism and risk of Sickle cell anemia (SCA). Therefore, we studied the association of miR-423 genotyping (rs6505162C>A) with susceptibility of sickle cell disease among Saudi Arabia population. Ferrara (1999) [17] reported that vascular endothelial growth factor (VEGF) is a mitogen for vascular endothelial cells derived from arteries, veins, and lymphatics. Lubin (1997) [18] reported that the interactions between vascular endothelium and sickle cells are essential events in many of the clinical complications including vaso-occlusive events (VOEs) of SCD. Solovey et al. [19] indicated that sickle cell anemia involves an abnormal and enhanced antiapoptotic tendency for endothelial cells and VEGF was responsible for this behavior. Bottomley et al. [20] reported that overexpression of VEGF prompts high expression levels of intercellular adhesion molecule-1 (ICAM-1) and high expression levels of VEGF and that was measured in the plasma of SCD patients. Several studies have reported that an abnormal adherent of RBCs to endothelial cells and their adhesiveness correlates with clinical severity in SCD patients [21]. In addition, there is an association between VEGFA gene variants, VEGF secretion with vaso-occlusive crisis (VOC) in SCD patients; therefore, it was concluded that the specific VEGFA variants could contribute to the pathogenesis of SCD with vaso-occlusive crisis VOC [22,23]. In light of this, we investigated the association of VEGF-2578 C>A genotyping with susceptibility of sickle cell disease in Saudi Arabia population.

Nowadays, many molecular methodologies have been utilized to detect gene polymorphism or mutations, such as Sanger sequencing, but this technique cannot rapidly screen large numbers of mutations in samples. Fortunately, this problem can be solved by massive next-generation sequencing (NGS) [24]. However, both Sanger sequencing and NGS are expensive. ARMS-PCR is based on the principle that the 3′-terminal nucleotides of the PCR primer must be complementary to its target sequence for efficient amplification. ARMS-PCR has been approved by the China Food and Drug Administration (CFDA) and has become a widely used method in clinical practice. Therefore, the aim of this study was to develop, optimize, and validate a direct T-ARMS-based PCR assay for the precise and rapid genotyping of vascular endothelial growth factor receptor and microRNA-423 gene abnormalities and their association with fetal hemoglobin expression in the patients with Sickle Cell Disease.

## 2. Materials and Methods

### 2.1. Selection Criteria of Patients

The study was performed on clinically confirmed cases of SCD and included 287 subjects, 127 of which were Sickle cell disease patients and 160 of which were healthy individuals. The SCD patients were diagnosed by HPLC. In addition, any patients with previous history of any chronic with disease were excluded from this study.

### 2.2. Sample Collection

From each clinically confirmed case of SCD patient, 3 mL of peripheral blood sample was collected by venipuncture in EDTA tubes. Healthy controls were enrolled from the general population of the same geographical region. Our study included one hundred sixty subjects visited King Khaled hospital for routine checkup and simple routine medical check-up was performed (such as complete blood count (CBC), Kidney Function Test, Liver Function Test etc.). A standard questionnaire was used and history of illness if detected was recorded by a health practitioner.

### 2.3. Genomic DNA Extraction

Genomic DNA was extracted from the whole blood samples using DNA extraction kit from Qiagen (Cat No. 69506, Hilden, Germany) according to the manufacturer’s instructions. Isolated DNA was dissolved in nuclease-free water and then stored at 4 °C until use. The purity of the obtained DNA was checked by running the sample in 1% agarose gel. The concentration of the extracted DNA was determined by absorbance at 260 nm and 280 nm using a NanoDrop™ (Thermo Scientific, Waltham, MA, USA).

### 2.4. Genotyping for microRNA-423 rs6505162 C>A and VEGF-2578 C>A

Optimization of an amplification-refractory mutation system PCR was performed by using tetra-primers specific for miR-423 rs6505162 C>T and VEGF-2578 C>A genotyping polymorphism. ARMS primers were designed by using Primer3 software online free software as represented in Table 1.

### 2.5. Allele Genotyping of miR-423-rs6505162 C>A

A PCR reaction was performed in a total volume of 12 µL consisting of template DNA (50 ng), FO—0.12 µL, RO—0.12 µL, FI—0.12 µL, RI—0.12 µL (25 pmol of each primer) and 6 µL from Green PCR Master Mix (2X) (K1081) (Thermo Scientific, Waltham, MA, USA). The final volume of 12 µL was adjusted by adding nuclease-free ddH_2_O. At the end, 2 µL of DNA was added from each patient.

PCR thermocycling conditions used were at 95 °C for 12 min followed by 40 cycles of 95 °C for 39 s, 62 °C for 42 s (miR-423 C>A genotyping), 58 °C for 40 s (VEGF-2578 C>A genotyping), 72 °C for 43 s followed by the final extension at 72 °C for 8 min.

**Figure 1:**
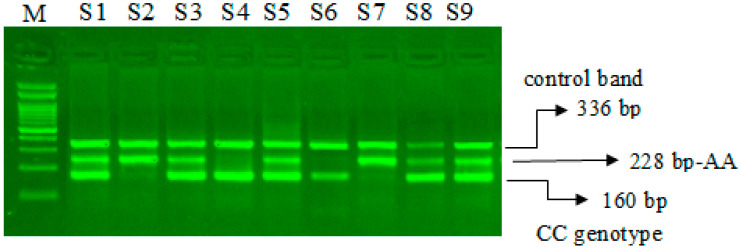
Optimization of Amplification-refractory mutation system (ARMS) primers for microRNA-423 rs6505162 C>A genotyping in SCD patients. M-100 bp DNA ladder, Heterozygous-(CA)-S1, S3, S5, S8, S9, Homozygous–(AA) S2, S7, Homozygous-(CC)-S4, S6.

**Figure 2:**
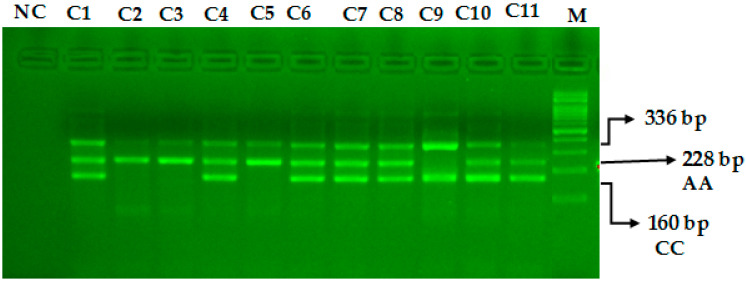
Optimization of Amplification-refractory mutation system (ARMS) primers for microRNA-423 rs6505162 C>A genotyping in healthy controls. M-100 BP DNA ladder, Heterozygous-(AC)-C1, C4, C6, C7, C8, C10, C11, Homozygous-(AA)-C2, C3, C5, Homozygous-(CC)-C9.

The miR-423-rs6505162 C>A PCR products were resolved through 2% agarose gel electrophoresis stained with Sybre safe dye. The gel image was visualized by gel documentation system from Bio-Rad. Outer primers FO and RO amplify the interested region of the miR-423-rs6505162 C>A genotyping gene site and generating a band of 336 bp and that serve as a control for DNA purity. Primers FI and RO amplify a C genotype generating a band of 160 bp, and primers FO and RI generate a band of 228 bp from the T genotype as shown in Figure 1 and Figure 2.

### 2.6. Allele Genotyping of VEGF-2578 C>A

**Figure 3:**
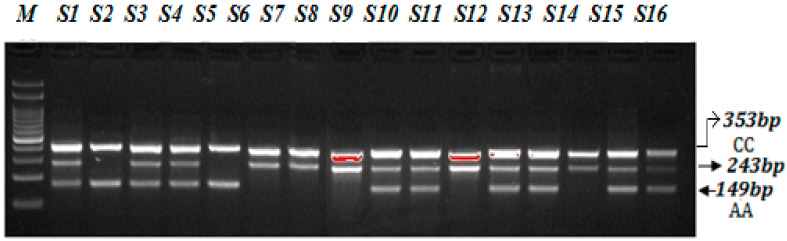
Optimization of Amplification-refractory mutation system (ARMS) primers for VEGF-2578 C>A genotyping in SCD patients. M-100 BP DNA ladder, Heterozygous-(AC)-S1, S3, S4, S9, S10, S12, S13, S15, S16, Homozygous-(AA)-S2, S5, Homozygous-(CC) S6, S7, S8, S11, S14.

A PCR reaction was performed in a total volume of 12 µL consisting of template DNA (50 ng), FO—0.10 µL, RO—0.10 µL, FI—0.10 µL, RI—0.1 µL (25 pmol of each primer) and 6 µL from Green PCR Master Mix (2X) (K1081) (Thermo Scientific, Waltham, MA, USA). The final volume of 12.50 µL was adjusted by adding nuclease-free ddH_2_O. At the end, 2.5 µL of DNA was added from each patient. The thermocycling conditions used were at 95 °C for 9 min followed by 30 cycles of 94 °C for 30 s, 58 °C for 35 s, 72 °C for 40 s followed by the final extension at 72 °C for 10 min. VEGF-2578 C>A gene amplification products were separated by electrophoresis through 2% agarose gel stained with 0.5 μg/mL ethidium bromide and visualized on a UV transilluminator. Primers FO and RO flank the exon of the VEGF-2578 C>A gene, resulting in a band of 353 bp to control for DNA quality and quantity. Primers Fwt and RO amplify a wild-type allele (C genotype), generating a band of 243 bp, and primers FO and Rmt generate a band of 149 bp from the mutant allele (A genotype) as depicted in the Figure 3.

### 2.7. Statistical Analysis

All statistical analyses were performed using SPSS26 (IBM Corp., 2017. IBM SPSS Statistics for Windows, Armonk, NY: IBM Corp). Deviations from Hardy-Weinberg disequilibrium (HWD) were calculated by a Chi-square (χ^2^) goodness-of-fit test. The associations between miR-423 (rs6505162 C>T) and VEGF-2578 C>A genotypes and SCD were estimated by computing the odds ratios (ORs), risk ratios (RRs) and risk differences (RDs) with 95% confidence intervals (CIs). A *p*-value < 0.05 was considered significant.

## 3. Results

### 3.1. Laboratory Characteristics of Patients with SCD

The various hematological tests were performed, and the results were documented from all 127 SCD patients. Few missing results were gathered from the medical records of SCD patients as described in Table 2. The mean percentage for different hemoglobin variants HbA1, HbA2 and HbF were calculated for all 127 SCD patients as 64.55%, 4.70% and 0.79%, respectively. The sickle cell hemoglobin (HbS) expression level was in a range from 26.00% to 84.40%, with overall mean value of 35.60%.

### 3.2. The Hardy-Weinberg Equilibrium Analysis

Deviations from Hardy-Weinberg disequilibrium (HWD) were calculated by a Chi-square (χ^2^) goodness-of-fit test. The distribution and frequency of genotypes as well as allele of the miR-423 rs6505162 C>A and VEGF-2578 C>A obeyed Hardy-Weinberg disequilibrium (HWD) (*p* = 0.83) (χ^2^ = 0.043, *p* = 0.83) in the control group. Only ten percent samples from the healthy control group were randomly selected to review the SNP results showing that the accuracy rate was more than 99%.

### 3.3. Statistical Comparisons between SCD Patients and Controls for microRNA-423 C>A Genotypes

The association of miR-423 rs6505162 C>A genotypes in SCD cases compared to healthy controls was statistically significant (*p* = 0.034) (Table 3). Higher frequency of miR-423-AA genotype was reported in SCD patients 42.51% than healthy controls (23.75%). However, 57.5% of miR-423-CA heterozygosity was observed among healthy controls compared to 48% SCD patients. Among the SCD patients the frequency of A allele (fA) was higher than the healthy controls (0.66 vs. 0.53) as depicted in Table 3.

### 3.4. Association of VEGF-2578 C>A Genotypes between SCD Patients and Controls

In SCD patients, the CC, CT and TT genotype frequencies were 41%, 47% and 12%, respectively, whereas in healthy controls CC, CT and TT genotype frequencies were 51%, 23% and 26%, respectively (Table 4). The distribution of VEGF-2578 C>A genotypes observed between SCD patients and healthy controls was significant (*p* < 0.013). Moreover, the frequency of C allele (fC) was found to be significantly higher among SCD patients than in healthy controls HC (0.65 vs. 0.50) (Table 4).

### 3.5. Multivariate Analysis of microRNA-423C>A Polymorphism between SCD Patients and Healthy Controls

An unconditional logistic regression was used to estimate associations between the genotypes and risk of Sickle cell disease patients (Table 5). It was found that an increased severity of Sickle cell disease patients was associated with the microRNA-423-AA genotype in an allele dosage-dependent manner.

Our results indicated that in the codominant model, the AA genotype of the microRNA-423 polymorphism was linked with increased SCD severity with OR 2.36 (95%) CI = (1.15–4.84), RR = 1.51 (1.09–2.09), *p* < 0.018. In case of dominant inheritance model, (CA + AA) vs. CC genotypes are not associated with SCD severity with OR = 1.47 (95%) CI (0.78–2.78), RR = 1.17 (0.91–1.51), *p* < 0.23 (Table 4). In case of the recessive model, (CC + CA) vs. (AA) genotypes were associated with increased Sickle cell disease severity with OR = 2.19 (95%) CI (1.32–3.62), RR = 1.46 (1.12–1.92), *p* < 0.002. The microRNA-423 AA allele is associated with SCD severity with OR = 1.57 (95%) CI (1.13–2.19), RR = 1.22 (1.05–1.41), *p* < 0.007) (Table 5) and may be considered to be genetic modifiers.

### 3.6. Association of VEGF-2578 C>A Gene Variation with SCD Susceptibility Utilizing Multivariate Analysis

A multivariate analysis based on logistic regression such as odds ratio (OD) and risk ratio (RR) with 95% confidence intervals (CI) were calculated for each group to estimate the association between VEGF-2578 C>A genotypes and risk to SCD and the data are summarized in Table 5. Our results indicated that in the codominant model, the VEGF-2578-CA genotype was strongly associated with increased SCD severity with OR 2.56 (95%) CI = (1.36 to 4.82) RR = 1.69 (1.16 to 2.45) *p* < 0.003 whereas VEGF-2578-AA genotype was not associated with SCD severity with OR 0.65 (95%) CI = (0.29 to 1.42), RR = 0.84 (0.63 to 1.130) *p* < 0.28. The VEGF-2578-A allele was not associated with Sickle cell disease severity with OR = 1.18 (95%) CI (0.77–1.81), RR = 1.08 (0.08–1.32), *p* < 0.44) (Table 6).

### 3.7. Association of HbA1, HbA2, HbF and HbS with miR-423 rs6505162 Genotypes in SCD Patients

**Figure 4:**
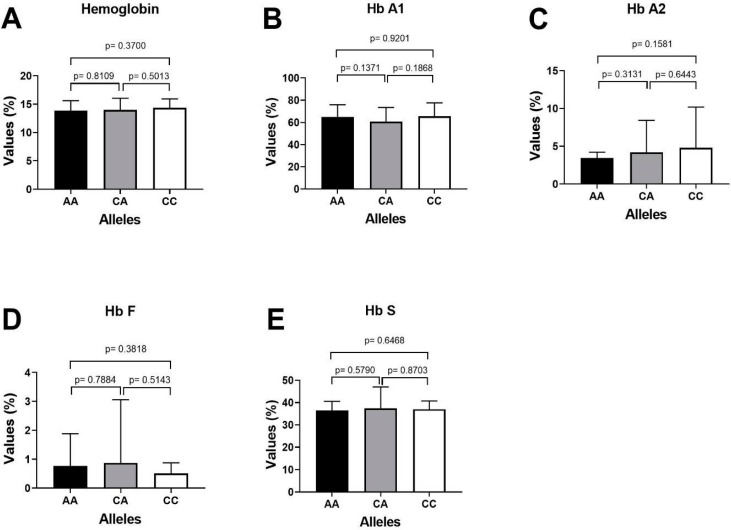
Clinical association of HbA1, HbA2, HbF and HbS with miR-423 genotypes in SCD patients. (**A**) Comparing *p* value of Hemoglobin with miR-423 genotypes (AA/CA/CCs). (**B**) Comparing the *p* value of HbA1 with miR-423 genotypes AA positive with AA negative samples. (**C**) Comparing the *p* value of HbA2 with miR-423 genotypes AA positive with AA negative samples. (**D**) Comparing the *p* value of HbF with miR-423 genotypes AA positive with AA negative samples. (**E**) Comparing the *p* value of HbS with miR-423 genotypes AA positive with AA negative samples.

The important laboratory characteristics HbA1, HbA2, HbF and HbS of patients with sickle cell disease were compared with the miR-423 C>A genotypes as depicted in Table 7. The higher expression of fetal hemoglobin HbF was reported in SCD patients carrying CA genotype of miR-423 rs6505162 CA-0.98%) followed by genotype CC-0.64% and AA-0.59%. Similarly, the patients (SCD) carrying miR-423 CC genotype, the mean % percentage expression level of different hemoglobin variants HbA1, HbA2, HbF and HbS were 64.53%, 3.36%, 0.64% and 35.6%, respectively, and Sickle cell disease patients carrying miR-423 AA genotype, the mean percentage expression level of different hemoglobin variants HbA1, HbA2, HbF and HbS were 65.9%, 4.40%, 0.59% and 36.1%, respectively (Table 7), and patients carrying miR-423 with heterozygosity genotype (miR-423 CA) the mean percentage expression level of different hemoglobin variants were HbA2, HbA1, HbF and HbS 3.74%, 59.98%, 0.98% and 38.1%, respectively. Clinical association of HbA1, HbA2, HbF and HbS with miR-423 genotypes in SCD patients is summarized in Figure 4.

## 4. Discussion

### 4.1. Role of ARMS-PCR for SNP Studies

MiRNAs are a group of non-coding RNAs of ~22 nucleotides in length. They post-transcriptionally control the expression of their target genes as well as chromatin-remodeling, differentiation, apoptosis, and proliferation. Decreased fetal hemoglobin in SCD patients is a big issue. Increasing fetal hemoglobin (HbF) is a significant therapeutic tool to overcome anemia and ineffective hematopoiesis. Different modalities including hydroxyurea, epigenetic modifications and microRNA-based regulation are used for induction of γ globin which may be used for therapeutic purposes in β-thalassemia and SCD patients. Recently it has been possible to increasing *γ globin* gene expression and fetal hemoglobin (HbF) production in these patients [25].

Although several sophisticated techniques and tools are applied to study genotyping and clinical diagnosis, but most of them are very complex, expensive for the laboratories in undeveloped countries. Previously, β-thalassemia prenatally was diagnosed by successfully using ARMS-PCR system in China and in Iran (Fu et al., and Moghadam et al.), respectively [26,27]; Chiu et al., utilized ARMS-PCR to detect the wild as well as mutant mitochondrial tDNA heteroplasmies [28], whereas ARMS-PCR was applied to the antenatal diagnosis of cystic fibrosis [29]. Rashid Mir et al., applied ARMS-PCR for studying genotyping in coronary artery disease, breast cancer and also utilized ARMS-PCR for microRNA genotyping [30,31,32,33]. Recently, Singh et al. [34] utilized ARMS PCR for prenatal diagnosis for SCD. Similarly, Aquino et al. [35] utilized ARMS PCR to detect known mutations of Cystic fibrosis transmembrane conductance regulator (CFTR) gene in Peruvian patients. ARMS-PCR and DNA sequencing was combined in East-Western Indian population for better management for the diagnosis of β-thalassemia [36]. Keeping all these studies in mind, it is recommended that ARMS-PCR may be utilized in the prenatal as well as postnatal diagnosis of various genetic diseases. Researchers in the past have used different techniques for genotyping of specific polymorphic nucleotide loci [37,38,39,40,41,42,43,44,45,46,47,48,49]. In this study we have successfully optimized the ARMS-PCR assay to study the genotyping of miR-423 and VEGF in SCD.

### 4.2. Steps for Optimization of ARMS-PCR Primers

The ARMS PCR is a powerful technique for detecting any mutation involving a single-base change. However, the optimization step required hard work and is time-consuming. For optimization of miR-423-rs6505162 C>T and VEGF-2578 C>A genotyping a gradient PCR was performed using different annealing temperatures and a lesser number of cycles were used (25 to 30 cycles). We made some small changes in the reagent concentrations, which significantly affected the PCR—especially MgCl2. Balancing the inner primers band was a key step. In order to balance the inner primers band, it was important to observe the band intensity and which band was the weakest in order to promote this band by increasing its concentration. This optimization for miR-423-rs6505162 C>T and VEGF-2578 C>A genotyping was achieved in a series of optimization experiments in a single run by the gradient PCR machine following some previous reported studies [21,22,23,24]. The annealing temperature via gradient PCR was optimized from 58 °C to 62 °C, but the best results were obtained at a temperature 62 °C for miR-423-rs6505162 C>T as depicted in Figure 1 and Figure 2. Similarly, the best results were obtained for VEGF-2578 C>A genotyping at a temperature 58 °C as depicted Figure 3. The use of our ARMS-PCR for of miR-423-rs6505162 C>T and VEGF-2578 C>A genotyping fulfils our goal. ARMS-PCR proved to be rapid, accurate, inexpensive and highly applicable.

### 4.3. Association of miR-423 rs6505162 C>A Genotypes and Fetal Hemoglobin in Sickle Cell Disease

Sickle cell disease (SCD) is a Mendelian disorder caused by a point mutation leading to a single amino acid substitution (Glu → Val) in the beta subunit of hemoglobin, the principal oxygen transporter in red blood cells. It has been estimated that SCD results in the annual loss of several millions of disability-adjusted life years, particularly in the developing world. Recent studies have shown that together, common SNPs at the BCL11A, HBS1L-MYB, and beta-globin (HBB) loci account for >20% of the variation in HbF levels in SCD patients and provide a clear example of inherited common sequence variants modifying the severity of a monogenic disease [49,50]. A large variety of regulatory factors that control the transcription of HbF has been discovered [51]. A number of studies have indicated towards the regulation of fetal hemoglobin being mediated by miRNAs. It was demonstrated post-transcriptional regulation of HU-mediated γ-globin expression through miRNA in SCD patients [52,53,54].

### 4.4. Hemoglobin Variables and microRNA-423 Genotypes

Fornari et al. [55] reported that the alterations in expression of microRNAs (miR-503/-144/-320/-451/-146/ etc.) in sickle cells or in thalassemic compared with normal RBCs may induce clinical severity these patients. Moreover, it is now proved that miRs are transcriptionally regulating erythroid-specific genes such as KLFs and Kruppel-like transcription factor D (*KLFD*) and therefore are involved in the regulation of expression of *globin* genes. Therefore, it can be argued that changes in expression of these small microRNAs are effective in reducing clinical complications in thalassemic patients. We studied the association of microRNA-423 with hemoglobin variables HbA1, HbA2, HbF and HbS of the patients with sickle cell disease were compared with the 3 genotypes (AA, AC and CC) of miR-423. The *p* values using unpaired *t*-test was calculated by comparing alleles with each other. In case of Sickle cell disease patient, those with miR-423 CC genotype, the mean percentage levels for HbA1, HbA2, HbF and HbS reported were 64.53%, 3.36%, 0.64% and 35.6%, respectively, whereas among SCD patients with miR-423 AA genotype, the mean percentage levels were 65.9%, 4.40%, 0.60% and 36.1%, respectively (Table 7). Similarly, it was observed that in case of SCD patients with miR-423 CA genotype, the mean percentage levels for HbA1, HbA2, HbF and HbS were 59.98%, 3.74%, 0.98% and 38.1%, respectively. 

Interestingly, HbS level was observed to be 65% in the patient who possessed both miR-423 AA and miR-423 CA genotypes. It was reported that an increased risk of Sickle cell disease severity was observed with miR-423 AA genotype. Byon et al. [56] reported that miR144/NRF2 regulatory mechanism predisposes HbSS to hemolysis, oxidative stress and more severe anemia. Therefore, erythrocyte microRNA expression manipulation provides a new approach to reduce clinical and pathological signs in SCD patients.

Duraisingh et al. [57] reported in his study that inhibition of the translocation of miR-451 and miR-223, by using 2′-O-methyl antisense oligonucleotides, reduced sickle cell resistance to malaria. This indicates that the induction of these microRNAs in infected cells can be used for host cell defense against pathogens. Frequency of miR-423 rs6505162 C>A genotypes in different population was determined as depicted in Table 8.

## 5. Conclusions

Collectively, our amplification-refractory mutation system (ARMS) has been proven to be rapid, accurate, sensitive, and inexpensive and is highly applicable for miR-423 rs6505162 and vascular endothelial growth factor gene variation VEGF-2578 C>A screening in laboratories and clinical practices. This research highlights the significance of elucidating genetic determinants that play roles in the amelioration of the HbF levels, that is used as an indicator of severity of clinical complications of the monogenic disease. Further well-designed studies with larger sample sizes are necessary to confirm our findings.

## Figures and Tables

**Table 1:** Amplification-refractory mutation system (ARMS) primers.

Direction	Primer Sequence	AT	Product Size
**Primer Sequence of miR-423 C>A Genotyping**			
miR-423 FO	5′-TTTTCCCGGATGGAAGCCCGAAGTTTGA-3′	62 °C	336 bp
miR-423 RO	5′-TTTTGCGGCAACGTATACCCCAATTTCC-3′		
miR-423FI (T allele)	5′-TGAGGCCCCTCAGTCTTGCTTCCCAA-3′		228 bp
miR-423 RI (C allele)	5′-CAAGCGGGGAGAAACTCAAGCGCGAGG-3′		160 bp
**Primer Sequence of VEGF-2578 C>A Genotyping**			
VEGF FO	5-CCTTTTCCTCATAAGGGCCTTAG-3	58 °C	353 bp
VEGF RO	5-AGGAAGCAGCTTGGAAAAATTC-3		
FI A VEGF (A allele)	5-TAGGCCAGACCCTGGCAA-3		149 bp
RI C VEGF (G allele)	5-GTCTGATTATCCACCCAGATCG-3		243 bp

**Table 2:** Laboratory characteristics of patients with SCD.

Variables	Mean ± SD	Range (Min–Max)
RBC (×10^12^/L)	5.14 ± 0.76	3.64–7.15
WBC (×10^9^/L)	8.18 ± 1.67	3.77–17.00
MCV (fL)	80.92 ± 6.07	66.01–92.01
Hematocrit (%)	40.96 ± 5.09	19.01–52.9
Hemoglobin (g/dL)	14.45 ± 1.88	5.90–18.25
Platelets (×10^9^/L)	351.92 ± 66.84	191.01–456.01
RDW (%)	12.73 ± 1.24	11.01–18.01
HbA1 (%)	63.3 ± 13.44	3.15–97.60
HbA2 (%)	3.36 ± 0.57	2.50–26.20
HbF (%)	0.64 ± 0.57	0.00–14.80
HbS (%)	35.6 ± 3.30	26.00–84.40

Hb—Hemoglobin, MCV—Mean corpuscular volume, RBC—Red blood cells, WBC—White blood cells and RDW-Red cell distribution width.

**Table 3:** Association of miR-423 rs6505162 C>A gene variation in SCD cases and controls.

Subjects	N	CC	CA	AA	Df Degree of Freedom	χ^2^ Chi Square	C	A	*p* Value
Cases	127	18 (14.17%)	61 (48%)	54 (42.51%)	2	6.74	0.34	0.66	0.034
Controls	160	30 (18.75%)	92 (57.5%)	38 (23.75%)			0.47	0.53	

**Table 4:** Association of VEGF-2578 C>A gene variation in SCD cases and controls.

Subjects	N	CC	CT	TT	Df	χ^2^	C	T	*p* Value
Cases	105	43 (41%)	49 (47%)	13 (12%)	2	6.13	0.65	0.35	0.013
Controls	105	54 (51%)	24 (23%)	25 (26%)			0.50	0.37	
		Df-degree of freedom	Chi square test χ^2^						

**Table 5:** Association of miR-423-rs6505162 C>A gene variation in SCD cases and controls.

Genotypes	Healthy Controls		SAD Cases		OR (95% CI)	Risk Ratio (RR)	*p*-Value
	(N = 160)	%	(N = 127)	**%**			
Codominant inheritance model							
miRNA-423-CC	30/160	18.75%	18/127	14.17%	1 (ref.)	1 (ref.)	
miRNA-423-CA	92/160	57.5%	69/127	54.33%	1.10 (0.566–2.15)	1.03 (0.80–1.34)	0.76
miRNA-423-AA	38/160	23.75%	58/127	45.67%	2.36 (1.15–4.84)	1.51 (1.090–2.09)	0.018
Dominant ant inheritance model							
miR-423-CC	30/160	18.75%	18/133	13.53%	1 (ref.)	1 (ref.)	
miR-423-(CA + AA)	130/160	81.25%	115/133	86.47%	1.47 (0.78–2.78)	1.17 (0.91–1.51)	0.23
Recessive ant inheritance model							
miR-423-(CC + CA)	122/160	76.25%	79/133	59.4%	1 (ref.)	1 (ref.)	
miR-423-AA	38/160	23.75%	54/133	40.6%	2.19 (1.32–3.62)	1.46 (1.12–1.92)	0.002
Allele							
miR-423-C	152/320	47.5%	97/266	36.5%	1 (ref.)	1 (ref.)	
miR-423-A	168/320	52%	169/266	63.5%	1.57 (1.13–2.19)	1.22 (1.05–1.41)	0.007

**Table 6:** Association of VEGF-2578 C>A gene variation in SCD cases and controls.

Genotypes	Healthy Controls	SCD Cases	OR (95% CI)	Risk Ratio (RR)	*p*-Value
	(N = 105)	(N = 105)			
Codominant ant inheritance model					
VEGF-2578-CC	54	43	1 (ref.)	1(ref.)	
VEGF-2578-CA	24	49	2.56 (1.36–4.82)	1.69 (1.16–2.45)	0.003
VEGF-2578-AA	25	13	0.65 (0.29–1.42)	0.84 (0.63–1.130)	0.28
Dominant ant inheritance model					
VEGF-2578-CC	54	43	1 (ref.)	1 (ref.)	
VEGF-2578 (CA + AA)	49	52	1.33 (0.76–2.33)	1.14 (0.87–1.50)	0.314
Recessive ant inheritance model					
VEGF-2578(CC + CA)	78	62	1 (ref.)	1 (ref.)	
VEGF-2578-AA	25	13	0.65 (0.30–1.38)	0.82 (0.63–1.07)	0.26
Allele					
VEGF-2578-C	132	105	1 (ref.)	1 (ref.)	
VEGF-2578-A	69	65	1.18 (0.77–1.81)	1.08 (0.88–1.32)	0.44

**Table 7:** HbA1, HbA2, HbF and HbS association with miR-423 rs6505162 genotypes in SCD patients.

MiR-423 Genotypes	HbA1 (Mean ± SD)	*p* Value	HbA2 (Mean ± SD)	*p* Value	HbF (Mean ± SD)	*p* Value	HbS (Mean ± SD)	*p* Value
CC (18)	63.3 ± 8.1	0.54	3.36 ± 0.57	0.45	0.64 ± 0.57	0.49	35.6 ± 3.3	0.35
CA (61)	59.8 ± 14.8		3.74 ± 2.90		0.98 ± 2.3		38.1 ± 11.0	
AA (48)	65.9 ± 11.8		4.40 ± 4.50		0.59 ± 0.95		36.1 ± 4.1	

**Table 8:** Frequency of miR-423 rs6505162 C>A genotypes in different population.

Country	Controls	CC	CA	AA	References
IRAN	300	141 (47%)	123 (41%)	36 (12%)	[45]
China	530	342 (64.53%)	170 (32.8%)	18 (3.40%)	[46]
Japan	623	412 (66.13%)	190 (30.5%)	21 (3.37%)	[47]
South America	807	284 (35%)	385 (48%)	138 (17%)	[48]
Australia	174	42 (24.14%)	80 (45.98%)	52 (29.89%)	[41]
South Africa	572	12 (2.1%)	184 (32.2%)	376 (65.7%)	[42]
Our study	160	30 (18.75%)	92 (57.5%)	38 (23.75%)	

## Data Availability

All the data associated with the current study has been presented in this manuscript.
